# Depletion of microglia exacerbates postischemic inflammation and brain injury

**DOI:** 10.1177/0271678X17694185

**Published:** 2017-01-01

**Authors:** Wei-Na Jin, Samuel Xiang-Yu Shi, Zhiguo Li, Minshu Li, Kristofer Wood, Rayna J Gonzales, Qiang Liu

**Affiliations:** 1 Department of Neurology, Tianjin Neurological Institute, Tianjin Medical University General Hospital, Tianjin, China; 2 Department of Neurology, Barrow Neurological Institute, St. Joseph’s Hospital and Medical Center, Phoenix, AZ, USA; 3 Department of Basic Medical Sciences, University of Arizona College of Medicine, Phoenix, AZ, USA

**Keywords:** Microglia, brain ischemia, colony-stimulating factor 1 receptor, neuroprotection

## Abstract

Brain ischemia elicits microglial activation and microglia survival depend on signaling through colony-stimulating factor 1 receptor (CSF1R). Although depletion of microglia has been linked to worse stroke outcomes, it remains unclear to what extent and by what mechanisms activated microglia influence ischemia-induced inflammation and injury in the brain. Using a mouse model of transient focal cerebral ischemia and reperfusion, we demonstrated that depletion of microglia via administration of the dual CSF1R/c-Kit inhibitor PLX3397 exacerbates neurodeficits and brain infarction. Depletion of microglia augmented the production of inflammatory mediators, leukocyte infiltration, and cell death during brain ischemia. Of note, microglial depletion-induced exacerbation of stroke severity did not solely depend on lymphocytes and monocytes. Importantly, depletion of microglia dramatically augmented the production of inflammatory mediators by astrocytes after brain ischemia*. In vitro* studies reveal that microglia restricted ischemia-induced astrocyte response and provided neuroprotective effects. Our findings suggest that neuroprotective effects of microglia may result, in part, from its inhibitory action on astrocyte response after ischemia.

## Introduction

Cerebral ischemia is a leading cause of mortality and disability.^1^ Despite recent advances of reperfusion therapy, only limited numbers of patients can benefit from these treatments.^2^ Together with the failure of many neuroprotectants, the scarcity of effective medications for ischemic brain injury prompts a reappraisal of the mechanisms behind the pathogenic processes triggered by brain ischemia and reperfusion.

Brain ischemia and reperfusion activate the immune system.^1,3–5^ The activated immune responses favor infarct growth and shape neurological outcomes.^5^ As brain resident cells, microglia are the first responders in response to ischemia and are engaged in intimate cross-talk with other intrinsic brain cells and infiltrating leukocytes from the periphery that enter the brain through the compromised blood–brain barrier.^6–8^ In response to brain ischemia, microglia promptly acquire properties of reactive species generation, antigen presentation, phagocytosis, and the production of inflammatory mediators including interleukin (IL)-1β, tumor necrosis factor (TNF)-α, IL-6, and matrix metalloproteinases.^7,9–12^ On the other hand, microglia also have an anti-inflammatory role by producing factors such as IL-4 and IL-10 during the resolution phase of brain inflammation, upon their return back to a surveillance state.^13,14^ These properties support microglia as active players in ischemic brain injury.^15–24^ However, the precise influence of microglia on brain inflammation and their contribution to ischemic brain injury has not been adequately studied.

As the most abundant cell type in the central nervous system (CNS), astrocytes constitute nearly 50% of brain volume. At the onset of brain injury, astrocytes actively participate in the production of pro-inflammatory factors such as C-C motif chemokine ligand 2, IL-1β, IL-6, etc., and thus they elicit potent pro-inflammatory properties.^25^ In addition, astrocytes are also considered as non-professional antigen presenting cells.^26,27^ Depending on the stage of brain pathology, astrocytes may acquire anti-inflammatory properties such as scar formation and restriction of inflammation by producing TGF-β.^28,29^ Evidence suggests that inhibition of astrocyte activity or function correlates with decreased infarct size. Treatments that decrease infarct size are often accompanied by attenuated astrocyte responses.^30,31^ These findings suggest that activation of astrocytes contributes to neural injury after brain ischemia.^30–33^ However, no studies have investigated the effects of microglia on astrocyte response in the setting of brain ischemia, and whether this process has any clinical significance.

The survival of microglia depends on colony stimulating factor 1 receptor (CSF1R) signaling.^34–38^ Reportedly, dietary treatment with a CSF1R/c-Kit inhibitor PLX3397 effectively depletes microglia in mice without apparent abnormalities in neurological function.^34,39^ PLX3397 treatment has no prominent impact on peripheral myeloid cells. As microglia are the major brain cell type that expresses CSF1R, PLX3397 treatment has minimal impact on either brain cell types such as neurons, astrocytes, and oligodendrocytes.^37,38^ Therefore, the use of PLX3397 offers an opportunity to determine whether and/or how microglia may impact brain inflammation and ischemic brain injury. In this study, we demonstrated that microglia elimination exacerbated brain inflammation and stroke severity. The neuroprotective effect of microglia may result from its restriction of the ischemia-induced astrocyte response.

## Materials and methods

### Animals

Male C57BL/6 (B6) mice and Rag2^−/−^γc^−/−^ mice were purchased from Taconic (Taconic Biosciences). The mutant mice were back-crossed to the B6 background for 12 generations. For all experiments, 6 - to 12-week-old, 23–25 g body weight, age-matched littermates were used. All mice were randomly assigned to experimental groups. Randomization was based on the random number generator function in Microsoft Excel 2013. Mice were housed under standardized light-dark cycle conditions with access to food and water ad libitum. All surgeries were performed under isoflurane anesthesia. Mice were housed in pathogen-free conditions at the animal facilities. All animal experiments were performed in accordance with the recommendations of the Guide for the Care and Use of Laboratory Animals of the National Institutes of Health and in accordance with the ARRIVE (Animal Research: Reporting In Vivo Experiments) guidelines. Animal studies were approved by the Animal Care and Use Committees of the Barrow Neurological Institute and Tianjin Neurological Institute.

### Compounds and treatment protocol

As previously described,^34^ PLX3397 (Selleckchem, Houston, TX) was formulated in AIN-76 A standard chow at 40 mg/kg/day for 21 consecutive days prior to middle cerebral artery occlusion (MCAO) surgery and continued until the end of experiments. AIN-76 A standard chow alone served as the control. Bindarit (2-methyl-2-[[1-(phenylmethyl)-1H-indazol-3-yl]methoxy] propanoic acid), an inhibitor of monocyte chemotactic protein synthesis, was diluted in 0.5% carboxymethylcellulose aqueous solution, and administered at a dose of 50 mg/kg by oral gavage, twice daily. Bindarit treatment was initiated immediately before MCAO and continued until the end of experiments. Control animals received an equal volume of carboxymethylcellulose.

### MCAO procedure

C57BL/6 (B6) or Rag2^−/−^γc^−/−^ mice (*Rag2/Il2rg* compound mutant mice, lack of lymphocytes) were subjected to focal cerebral ischemia produced by transient intraluminal occlusion of the middle cerebral artery (MCA) using a filament method as previously described.^40–43^ Briefly, MCAO was performed under anesthesia by inhalation of 3.5% isoflurane and maintained by inhalation of 1.0–2.0% isoflurane in 70% N_2_O and 30% O_2_ by a nose cone. Cerebral blood flow (CBF) was monitored for 5 min both before and after MCAO, and immediately before and after reperfusion with a laser Doppler probe (model P10, Moor Instruments, Wilmington, DE). A monofilament made of 6–0 nylon with rounded tip was used to induce focal cerebral ischemia. After 60 min of MCAO, the occluding filament was withdrawn gently back into the common carotid artery to allow reperfusion. Thereafter, CBF was monitored for an additional 10 min before the incision site was sutured, and mice were allowed to recover from anesthesia. Sham control mice were subjected to the same surgical procedure, but the filament was not advanced far enough to occlude the MCA. Mice that had a residual CBF <20% of preischemic levels throughout the ischemic period and CBF recovery >50% within 10 min of reperfusion were used in the study. Among the total of 294 mice used in this study, 14 mice were excluded due to death after surgery, and 18 mice were excluded due to inadequate reperfusion. 7T-MRI was used to determine infarct volume at 24 h or 72 h after MCAO (see Neuroimaging).

### Neurological assessment

Neurological deficit assessment was performed by experimenters blinded to the sham and MCAO groups as we previously described.^40–44^ The modified Neurological Severity Score (mNSS) test consisted of motor, sensory, reflex, and balance assessments with the highest possible score being 18. The rating scale was as follows: A score of 13–18 indicates severe injury, 7–12 indicates moderate injury, and 1–6 indicates mild injury. Following surgery, each mouse was assessed on a scale from 0 to 18 after recovery from the MCAO surgical procedure. Mice with score <6 or above a score of 13 at 24 h post MCAO (prior to treatment) were not included in the study. In all experiments, nine mice were excluded due to criteria limitations set for the mNSS scoring system.

Corner turning test was used to assess sensorimotor and postural asymmetries. All mice tested were allowed to enter a corner with an angle of 30 degrees which required the subject to turn either to the left or the right to exit the corner. This was repeated and recorded 10 times, with at least 30 s between trials, and the percentage of right turns out of total turns was calculated. The ability of a mouse to respond to a vibrissae-elicited excitation by forward moving of its forelimb was evaluated with the forelimb placing test, as previously described.^45^ Briefly, animals held by their trunk, were positioned parallel to a table top and slowly moved up and down, allowing the vibrissae on one side of the head to brush along the table surface. Refractory placements of the impaired (left) forelimb were evaluated and a score was calculated as number of successful forelimb placements out of 10 consecutive trials.

### Neuroimaging

MRI scans were performed using a 7 T small animal, 30-cm horizontal-bore magnet and BioSpec Avance III spectrometer (Bruker, Billerica, MA). A 72 mm linear transmitter coil and mouse surface receiver coil were used for mouse brain imaging as we previously described.^40,41,43,44,46^ Mice were under anesthesia by inhalation of 3.5% isoflurane and maintained by inhalation of 1.0–2.0% isoflurane in 70% N_2_O and 30% O_2_ by a nose cone. During MRI scan, the animal’s respiration was continually monitored by a small animal monitoring and gating system (SA Instruments, Stoney Brook, NY) via a pillow sensor positioned under the abdomen. Mice were placed on a heated circulating water blanket to maintain normal body temperature (36–37℃). Axial 2D multi-slice T2-weighted images of the brain were acquired with fat-suppressed rapid acquisition with relaxation enhancement (RARE) sequence (TR = 4000 ms, effective TE = 60 ms, number of average = 4, FOV = 19.2 mm × 19.2 mm, matrix size = 192 × 192, slice thickness = 0.5 mm, TA = 6 m 24 s). The MRI data were analyzed with Image J software (National Institutes of Health) as we previously reported.^40–42,47^

To detect reactive oxygen species (ROS) generation in the brain, live bioluminescence images were captured using the Xenogen IVIS200 imager (Caliper LifeSciences, Hopkinton, MA).^40,44,48^ ROS production was assessed at 24 h after MCAO using luminol as we and others previously described.^42,48^ Briefly, mice were injected i.p. with 200 mg/kg luminol (Invitrogen, Carlsbad, CA,). At 10 min after luminol injection, bioluminescence images were captured. A region of interest tool was used to define and measure the chemiluminescent intensity within the brain. Dihydrolipoic acid (DHLA), a reduced form of α-lipoic acid which can scavange ROS, was used to confirm that the observed luminol signal was indeed due to ROS in vivo (Supplementary Figure 1). Data were collected as photons per second per cm^2^ using Living Image software (Caliper Life Sciences, Hopkinton, MA).

### Primary cell culture and oxygen-glucose deprivation

Primary neuron and glial cells culture were performed as described previously.^41^ Briefly, the loosely adhering microglial cells were isolated from a primary culture of mixed glial cells by shaking at 180 r/min for 30 min. Then the flask continued to shake at 240 r/min for 6 h to remove oligodendrocyte precursor cells in order to enrich the astrocytes. Isolated microglia and astrocytes were maintained in Dulbecco’s modified essential medium (DMEM) supplemented with glucose (4.5 g/L), L-glutamine, and pyridoxine, as well as 10% (v/v) heat-inactivated, characterized fetal bovine serum (Hyclone, Logan, UT), with medium changes every two to three days. The purity of microglial cultures as well as astrocytes was evaluated by staining as described for the microglia marker Iba1, and astrocyte marker GFAP to identify microglia and astrocytes.

Primary cortical neurons were seeded onto 35 mm culture dishes with 5 × 10^4^/cm^2^ cells and cultured at 37℃ in a CO_2_ incubator supplied with hepa-filtered room air. After eight days, the cultured dishes were separated into two groups, one for OGD chamber and one as control. The OGD group of neurons was individually treated with OGD pre-conditioned glial cell culture media for 24 h, including isolated microglial cell culture media, astrocyte culture media, and mixed glial cell culture media (microglia/astrocytes). Before OGD, all dishes were washed twice with glucose-free 1 × EBSS (Earle's Balanced Salt Solution). For OGD, the cells were transferred to the chamber containing 5% CO_2_ and 95% N_2_ for 30 min (for glial cells) or 5 min (for neurons) at 37℃. The control plates of cells were maintained in culture media with glucose in the regular oxygen-containing incubator.

### Immunostaining

The immunostaining was performed as we previously described.^40–44,49,50^ Briefly, mice were euthanized and whole brain removed, fixed in 4% paraformaldehyde, and then dehydrated with 15% and 30% sucrose. Whole brain was embedded in OCT for preparation of frozen sections. The frozen slices of 30 µm thickness were blocked in 5% goat serum for 1 h at room temperature. Thereafter, tissue sections were incubated with anti-mouse NeuN (1:100, ab177487, Abcam, Cambridge, MA) primary antibody at 4℃ overnight, and then incubated with FITC-conjugated goat anti-rabbit secondary antibodies (1:2000; BD Bioscience) at room temperature for 1 h. The cultured primary neurons were fixed in phosphate-buffered saline (PBS) containing 4% paraformaldehyde. After being washed in PBS, cells were permeabilized with 0.1% Triton X-100. Primary neurons were incubated with anti-mouse beta III Tubulin (1:100, ab78078, Abcam, Cambridge, MA) at 4℃ overnight, and then incubated with FITC-conjugated goat anti-mouse secondary antibodies (1:2000; BD Bioscience, San Jose, CA) at room temperature for 1 h. Nuclei were co-stained with 4′,6-diamidino-2-phenylindole (DAPI; Abcam, Cambridge, MA). In situ cell apoptosis of brain tissue or cultured neurons were detected by using terminal deoxynucleotidyl transferase dUTP nick end labeling (TUNEL, in situ BrdU-Red DNA Fragmentation assay Kit, ab66110, Abcam, Cambridge, MA) according to the manufacturer's protocol.^51^ Images were captured by fluorescence microscopy (Olympus, model BX-61). Image analysis was performed using Image J software (National Institutes of Health).

### Flow cytometry

Quantitative analysis of immune cell subsets or cytokines was prepared from spleen or brain tissues and stained with fluorochrome-conjugated antibodies as described.^40,44,46^ For in vitro experiments, 0.25% trypsin (Sigma-Aldrich, St. Louis, MO) was used to digest the cultured primary glial cells and cells were stained with fluorochrome-conjugated antibodies. Antibodies were labeled with one of the following fluorescent tags: fluorescein isothiocyanate (FITC), phycoerythrin (PE), PerCP-Cy5.5, allophycocyanin (APC), PE-Cy7 or APC-Cy7. The following antibodies were used: CD3 (145-2C11, 553066, BD Biosciences, San Jose, CA), CD4 (RM4-5, 552775, BD Biosciences, San Jose, CA), CD8 (53-6.7, 557654, BD Biosciences, San Jose, CA), NK1.1 (PK136, 551114, BD Biosciences, San Jose, CA), CD45 (30-F11, 12-0451-83, eBioscience, San Diego, CA), CD11b (M1/70, 25-0112-82, eBioscience, San Diego, CA), F4/80 (BM8, 123119, Biolegend, San Diego, CA), Ly6G (1A8, 127614, Biolegend, San Diego, CA), B220 (RA3-6B2, 553093, BD Biosciences, San Jose, CA), IL-1α (559810, BD Biosciences, San Jose, CA), IL-1β (NJTEN3, 12-7114-82, eBioscience, San Diego, CA), IL-6 (MP5-20F3, 12-7061-81, eBioscience, San Diego, CA), MCP-1 (2H5, 505903, Biolegend, San Diego, CA), TNFα (MP6-XT22, 557719, BD Biosciences, San Jose, CA), GFAP (GA5, 50-9892-82, eBioscience, San Diego, CA), iNOS (CXNFT, 53-5920-80, eBioscience, San Diego, CA). Flow cytometric measurements were performed on a FACSAria (BD Biosciences, San Jose, CA) and analyzed using FACSDiva and Flowjo 7.6 software (Informer Technologies, Ashland, OR).

### Cytokine array

Cytokines, chemokines, and growth factors in brain homogenates were analyzed by Proteome Profiler Mouse XL Cytokine Array. Brain homogenates were prepared from sham control and MCAO mice treated with or without PLX3397 24 h after MCAO. After the total protein concentration was adjusted to 1 mg/mL, cytokine levels in these samples were detected using a Mouse XL Cytokines Array Kit (R&D Systems Inc. Minneapolis, MN) according to the manufacturer’s instructions.

### Lactate dehydrogenase release

OGD-induced neuronal damage was assessed by measurement of LDH released into the media. Primary cultured neurons were individually treated with OGD pre-conditioned glial cell culture media for 24 h. At the end of treatment, 50 µL of primary cultured neuronal medium solution was transferred to a 96-well plate mixed with 50 µL of the LDH substrate, and the LDH reaction was performed using Pierce LDH Cytotoxicity Assay Kit (Thermo Scientific, Waltham, MA) following the manufacturer's instructions. After incubation for 0.5 h at room temperature, the reaction was ceased using stop buffer. To determine LDH activity, the absorbance at 680 nm (background signal) was subtracted from the absorbance at 490 nm. LDH release was expressed by normalizing the released LDH in the medium to the total LDH (released +intracellular).

### Statistical analysis

Power analysis and sample size calculations were performed using SAS 9.1 software (SAS Institute Inc. Cary, NC). Sample size calculation was performed at a power of 0.8 and to a significance level of 0.05. The experimental design was based on previous publications with similar mechanistic studies.^40,44,46,52,53^ Animals were randomly assigned to treatment condition. All results were analyzed by investigators blinded to the treatment. Statistical analyses were performed using GraphPad Prism 6.0 software. Two-tailed unpaired Student *t*-test was used to determine the significance of differences between two groups. One-way ANOVA followed by Tukey post hoc test was used for comparisons of three or more groups. Two-way ANOVA followed by Bonferroni post-tests was used for multiple comparisons. Significance was set at *P* < 0.05. Data are shown as Means ± SD.

## Results

### PLX3397 treatment eliminates microglia and exacerbates ischemic brain injury

**Figure 1.**
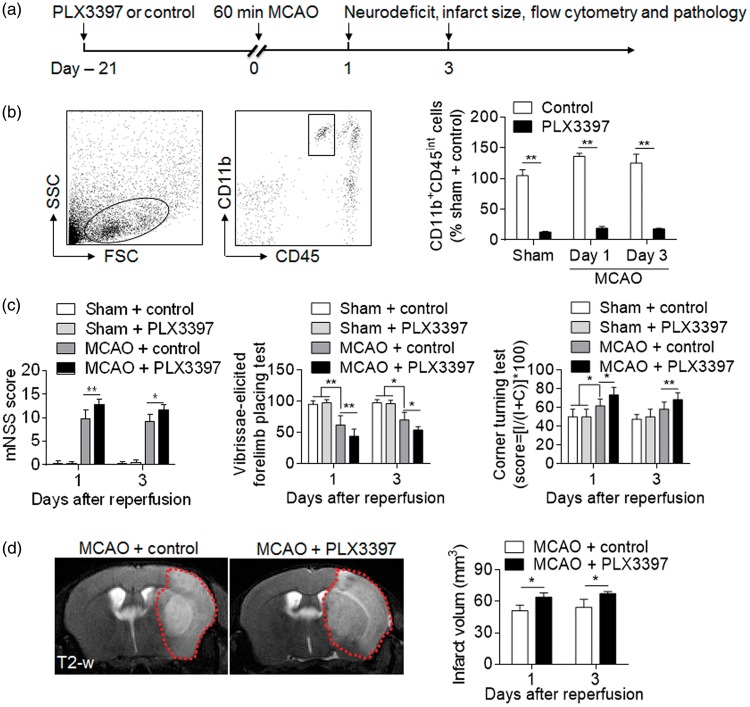
PLX3397 treatment eliminated microglia and exacerbated stroke severity in MCAO mice. Wild type C57BL/6 mice were fed PLX3397 or control chow diet for 21 days prior to sham or MCAO surgeries. After surgeries, these mice continued to receive PLX3397 or control diet until they were sacrificed. At day 1 or day 3 after 60 min MCAO and reperfusion, mice were subjected to neurological assessment, MRI, flow cytometry, and pathology staining. (a) Schematic showing the experimental design. (b) Representative flow cytometry plots show the gating strategy of CD11b^+^CD45^int^ microglial cell population. Bar graphs summarize the results from sham or MCAO mice receiving the indicated treatment at day 1 and day 3 after MCAO reperfusion. (c) Bar graphs illustrate the indicated neurological assessments of sham and MCAO mice receiving PLX3397 or control diet at day 1 or day 3 after reperfusion. (d) Representative MRI images show infarct area (outlined in red) in MCAO mice receiving PLX3397 or control diet after MCAO reperfusion. Bar graphs show the infarct volume of MCAO mice receiving PLX3397 or control diet at indicated time points. n = 15 mice per group. Error bars represent SD; *P < 0.05; **P < 0.01.

Wild type C57BL/6 mice were fed with PLX3397 or control diet for 21 days prior to sham or MCAO procedures (Figure 1(a)). Thereafter, these mice continued to receive PLX3397 or control diet until the end of experiments. At day 1 or day 3 after MCAO and reperfusion, the efficacy of microglia elimination by PLX3397 was determined, compared with sham operated mice fed with PLX3397 or control diet. As shown in Figure 1(b), flow cytometry analysis revealed that PLX3397 administration produced a reduction of nearly 90% of microglia (CD11b^+^CD45^int^) in brains of sham and MCAO mice. In contrast, PLX3397 treatment did not significantly alter neurological function in sham control (Figure 1(c)). Together, these data demonstrate the efficacy of microglial elimination by PLX3397 and indicate that PLX3397-induced depletion of microglia is independent of brain ischemia.

To determine the impact of microglial elimination on ischemic brain injury, we examined neurodeficits and infarct size in MCAO mice receiving PLX3397 or control diet. At day 1 or day 3 post ischemia and reperfusion, microglial elimination resulted in aggravated neurodeficits and enlarged infarct size (Figure 1(c) and (d)). These results show that microglial elimination exacerbated ischemic brain injury.

### Depletion of microglia enhances brain inflammation and cell death after brain ischemia

**Figure 2.**
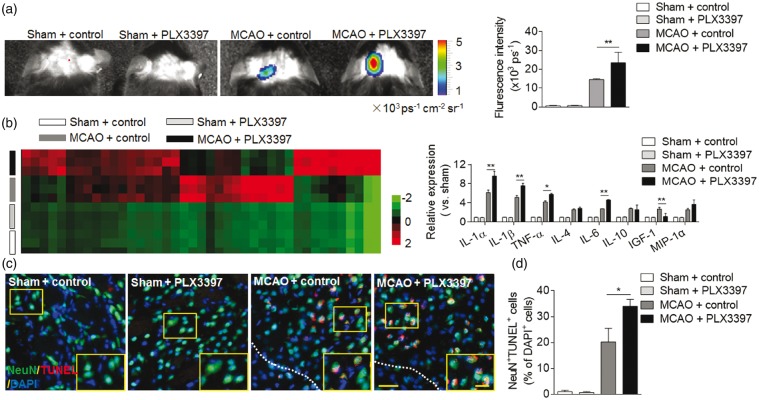
Depletion of microglia augmented brain inflammation and cell death after ischemia. C57BL/6 mice were fed with PLX3397 or control diet for 21 days prior to 60 min MCAO. After MCAO, mice continued to receive PLX3397 or control chow diet until they were sacrificed. (a) At 24 h after ischemia and reperfusion, representative bioluminescence images and quantification analysis show ROS generation in sham and MCAO mice receiving PLX3397 or control diet. n = 15 mice per group. (b) At 24 h after ischemia and reperfusion, brain tissues were obtained from MCAO mice receiving PLX3397 or control diet. Brain tissues from sham operated mice receiving PLX3397 or control diet were used as control. Brain homogenates were analyzed by a Mouse XL Cytokines Array kit. Heat map and cluster analysis show the expression of cytokines, chemokines, and growth factors in brain homogenates from sham and MCAO mice with indicated treatments. A heat map was generated and the relative pixel intensity of spots signal is indicated by the representative color code (red, higher; green, lower). Bar graphs show the significantly altered factors. n = 8 mice per group. (c) Images show immunostaining of neuron (NeuN; green) terminal deoxynucleotide transferase–mediated deoxyuridine triphosphate nick-end labeling (TUNEL; red) and 4′,6-diamidino-2-phenylindole (DAPI; blue) in brain sections from sham and MCAO mice treated with PLX3397 or control at 24 h after ischemia and reperfusion. Representative images of four independent experiments with five mice per group each are shown. Scale bars: 40 µm; 20 µm (inset). (d) Quantification of the percentage of NeuN^+^TUNEL^+^ cells in the sham mice and peri-infarct of MCAO mice treated with PLX3397 or control at 24 h after ischemia and reperfusion. Error bars represent SD; *P < 0.05; **P < 0.01.

We sought to understand the influence of microglial elimination on brain inflammation. As shown in Figure 2(a), ROS (denoting areas of inflammation) levels were significantly higher of was noted in MCAO mice fed with PLX3397 diet as compared to those fed with control diets. In contrast, there was no difference in the production of ROS in sham mice receiving PLX3397 treatment (Figure 2(a)). Using a Proteome Profiler Mouse XL Cytokine Array, we assessed the expression of cytokines, chemokines, and growth factors in the brain homogenates of MCAO mice after microglial elimination. At 24 h after MCAO, we found that PLX3397 treatment significantly upregulated the expression of pro-inflammatory cytokines including IL-1α, IL-1β, IL-6 and TNF-α, while the levels of growth factors such as IGF-1 were down-regulated (Figure 2(b)). In the determination of cell death after ischemia and reperfusion, we quantified TUNEL^+^ cells in brain sections from MCAO mice. PLX3397 treatment significantly increased the counts of NeuN^+^TUNEL^+^ cells in the peri-infarct regions (Figure 2(c) and (d)). These data revealed a profound role of microglia in the modulation of brain inflammation and neuronal death after ischemia.

### Depletion of microglia promotes leukocyte infiltration after brain ischemia

**Figure 3.**
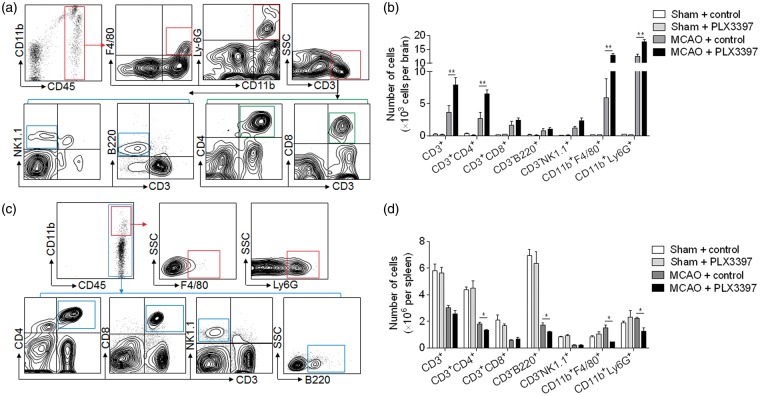
Depletion of microglia enhanced leukocytes infiltration after MCAO. Wild type C57BL/6 mice were fed with PLX3397 or control diet for 21 days prior to MCAO. At 24 h after ischemia and reperfusion, single cell suspensions were prepared from brain or spleen tissues of MCAO mice receiving PLX3397 or control diet. (a) Representative flow cytometry plots show the gating strategy of leukocyte subpopulations isolated from brain tissues. Plots show gating of T cells (CD45^high^CD3^+^), CD4^+^ T cells (CD45^high^CD3^+^CD4^+^), CD8^+^ T cells (CD45^high^CD3^+^CD8^+^), B cells (CD45^high^CD3^-^B220^+^), NK cells (CD45^high^CD3^-^NK1.1^+^), macrophages (CD45^high^CD11b^+^F4/80^+^) and neutrophils (CD45^high^CD11b^+^ Ly-6 G^+^). (b) Quantification of CNS-infiltrating lymphocytes, macrophages and neutrophils from sham and MCAO mice receiving the indicated treatments at 24 h after ischemia and reperfusion. (c) Representative flow cytometry plots show the gating strategy of leukocyte subpopulations isolated from the spleen. (d) Quantification of lymphocytes, macrophages and neutrophils in the spleen of sham and MCAO mice receiving indicated treatments at 24 h after ischemia and reperfusion. n = 15 mice per group. Error bars represent SD; *P < 0.05; **P < 0.01.

Infiltrating leukocytes are a prominent contributor to brain inflammation after ischemia. To examine the impact of microglial elimination on leukocyte infiltration, we quantified the numbers of brain-infiltrating leukocytes in MCAO mice fed with PLX3397 or control diets. PLX3397 treatment had no significant impact on immune cell infiltration in brains from sham mice (Figure 3). At 24 h after MCAO and reperfusion, we found that PLX3397 treatment increased the counts of brain-infiltrating neutrophils, macrophages, CD4^+^ T, and NK cells (Figure 3(a) and (b)). Moreover, the increase of brain-infiltrating leukocyte subsets was accompanied with a decrease of corresponding leukocyte subsets in the spleen (Figure 3(c) and (d)).

**Figure 4.**
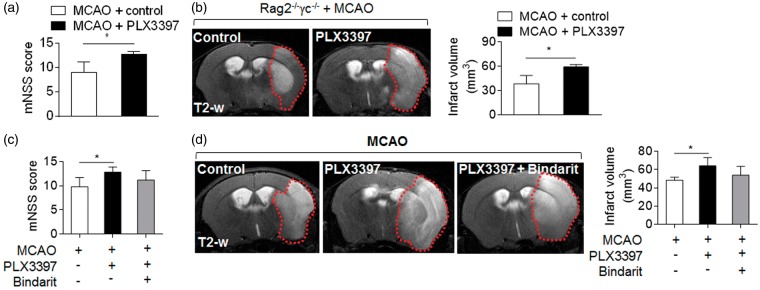
Depletion of microglia-induced exacerbation of stroke severity did not entirely depend on lymphocyte and monocyte infiltration. (a)–(b) Rag2^−/−^γc^−/−^ mice were fed with PLX3397 or control diet for 21 days prior to MCAO. After MCAO, these mice continued to receive PLX3397 or control diet until they were sacrificed. Bar graphs illustrate neurological scores of Rag2^−/−^γc^−/−^ mice receiving PLX3397 or control diet at 24 h after MCAO (a). n = 15 mice per group. MRI images show infarct area (outlined in red) in mice subjected to MCAO and indicated treatments at 24 h after reperfusion. Bar graphs illustrate the infarct volume of Rag2^−/−^γc^−/−^ mice receiving PLX3397 or control diet at 24 h after MCAO (b). n = 15 mice per group. (c)–(d) C57BL/6 mice were fed with PLX3397 or control diet for 21 days prior to 60 min MCAO. Starting from immediately after MCAO, bindarit (an inhibitor of monocyte chemotactic protein synthesis) was given orally at a dose of 50 mg/kg twice a day. MCAO mice receiving an equal volume of vehicle carboxymethylcellulose were used as controls. Bar graphs illustrate the infarct size of MCAO mice receiving indicated treatments at 24 h after ischemia and reperfusion (c). n = 15 mice per group. Representative MRI images show the infarct area (outlined in red) in MCAO mice receiving the indicated treatments at 24 h after ischemia and reperfusion. Bar graphs show the infarct volume of MCAO mice receiving indicated treatments at 24 h after ischemia and reperfusion (d). n = 15 mice per group. Error bars represent SD; *P < 0.05.

To understand to what extent the increase of brain-infiltrating immune cells may contribute to microglia depletion-induced exacerbation of stroke severity, we determined the effect of microglia depletion on ischemic brain injury using lymphocyte deficient Rag2^−/−^γc^−/−^ mice (lack of T, B, NK and NKT lymphocytes). Of interest, microglia depletion significantly aggravated neurodeficits and brain infarct volume in Rag2^−/−^γc^−/−^ mice (Figure 4(a) and (b)), suggesting that microglia depletion-induced aggravation of ischemic brain injury does not entirely depend on lymphocytes.

In addition to lymphocytes, the increase of brain-infiltrating monocytes may also contribute to brain injury. Therefore, we next examined whether monocytes contribute to microglia depletion-induced aggravation of ischemic brain injury. As monocyte chemoattractant protein 1 (MCP1, CCL2) is a key chemokine to promote monocyte recruitment, the use of an inhibitor of monocyte chemotactic protein synthesis, bindarit, has been shown to effectively block the homing of monocytes into the brain.^54^ We found that blockade of monocyte recruitment did not significantly affect microglia depletion-induced aggravation of ischemic brain injury (Figure 4(c) and (d)). Together, our data suggest that microglia depletion-induced aggravation of stroke severity may not entirely depend on the infiltration of periphery lymphocytes and monocytes.

### Microglia restricted ischemia-induced astrocyte response

**Figure 5.**
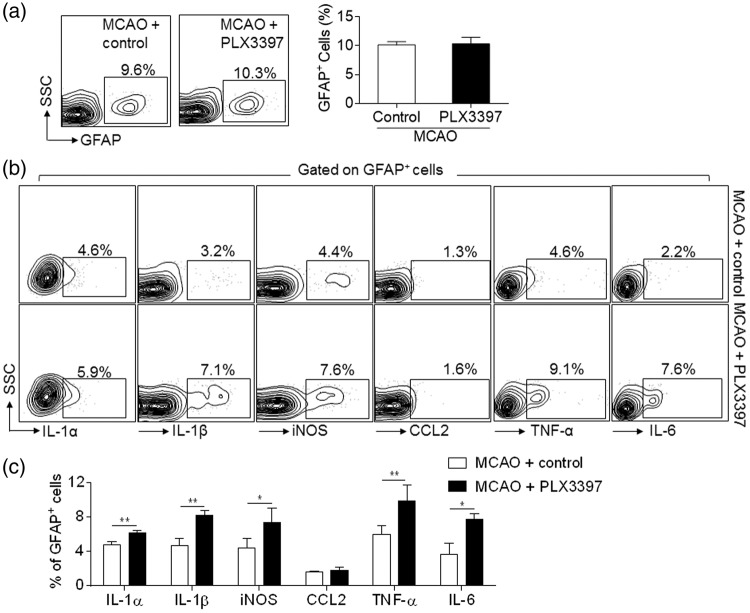
Depletion of microglia enhanced astrocyte response after brain ischemia. C57BL/6 mice were fed with PLX3397 or control diet for 21 days prior to MCAO. At 24 h after ischemia and reperfusion, single cell suspensions were prepared from brain or spleen tissues of MCAO mice receiving PLX3397 or control diet. (a) Representative flow cytometry plots and bar graph show astrocytes (GFAP^+^) in MCAO mice receiving PLX3397 or control diet, at 24 h after ischemia and reperfusion. (b)–(c) Representative plots (b) and bar graphs (c) of flow cytometry analysis show the expression of IL-1α, IL-1β, iNOS, CCL2, TNF-α, IL-6 in astrocytes obtained at 24 h after MCAO from mice receiving PLX3397 or control diet. n = 15 mice per group. Error bars represent SD; *P < 0.05; **P < 0.01.

Astrocytes are the most abundant cell type in the CNS and possess a potent pro-inflammatory function after brain injury. We therefore measured astrocyte response after MCAO and reperfusion in mice fed with PLX3397 or control chow diet. PLX3397 treatment did not affect the counts of astrocytes (Figure 5(a)). Of note, PLX3397 treatment led to a dramatic up-regulation of IL-1α, IL-1β, inducible nitric oxide synthase (iNOS), TNF-α and IL-6 in astrocytes after MCAO and reperfusion (Figure 5(b) and (c)), demonstrating a significant enhancement of astrocyte response after microglia depletion.

**Figure 6.**
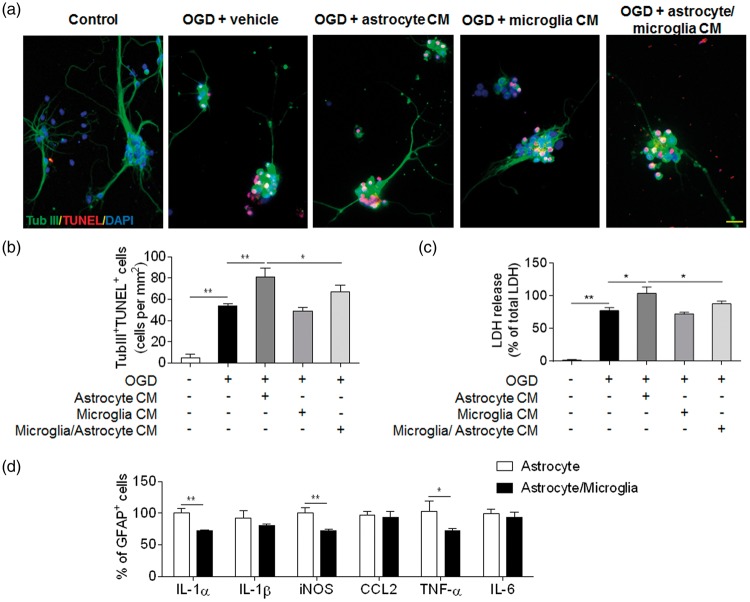
Microglia restricted ischemia-induced astrocyte response and neural injury. Primary cortical neurons were subjected to 5 min OGD. Immediately after OGD, culture media was replaced with regular neurobasal medium mixed with equal volume of glia-conditioned medium for 24 h. Glia-conditioned medium was obtained from cultured microglia, astrocytes or mixed microglia and astrocytes (microglia/astrocyte) at 24 h after 30 min OGD. (a) Representative images show staining of beta III Tubulin (green), DAPI (blue) and TUNEL (red) in primary cortical neurons exposed to OGD and glia-conditioned medium as indicated. Primary cortical neurons without exposure to OGD or glia-conditioned medium were used as controls. Scale bars: 20 µm. (b) Quantification of TUNEL^+^ cells after exposure to indicated treatment. CM: conditional medium. (c) Bar graphs show the level of released LDH from primary cortical neurons after exposure to indicated treatment. LDH release was expressed by normalizing the released LDH in the medium to the total LDH (released + intracellular). CM: conditional medium. (d) Primary astrocytes were cultured separately or together with microglia (microglia/astrocyte). After 30 min OGD and subsequent 24 h recovery, intracellular staining of GFAP and indicated cytokines was performed and analyzed by flow cytometry. Bar graphs show the percentage of GFAP^+^ cells expressing IL-1α, IL-1β, iNOS, CCL2, TNF-α and IL-6. Error bars represent SD.; *P < 0.05; **P < 0.01.

We asked whether the augmented astrocyte response may contribute to microglia depletion-induced aggravation of stroke severity. To answer this question, we compared the effect of conditioned media from cultured astrocytes, microglia, or mixed astrocyte/microglia after OGD on OGD-treated primary cortical neurons. We show that conditioned media from OGD-treated astrocytes alone augmented neuronal death, as manifested by increased counts of TUNEL^+^ cells and LDH release (Figure 6(a) to (c)). In contrast, conditioned media from OGD-treated microglia alone did not significantly affect OGD-induced neuronal death. Notably, conditioned media from cultures containing microglia plus astrocytes has reduced neural injury, as compared to neurons exposed to conditioned media from OGD-treated astrocytes (Figure 6(a) to (c)). In addition, the counts of astrocytes producing inflammatory mediators (IL-1α, iNOS and TNF-α) were significantly reduced when co-cultured with microglia (Figure 6(d)). These data support the findings that microglia can limit the astrocyte response after OGD insult, suggesting that the inhibitory action of microglia on astrocyte responses may contribute to the neuroprotective effect of microglia after brain ischemia.

## Discussion

This study provides evidence that microglia confers protection in ischemic brain injury. Elimination of microglia exacerbated brain infarction and neurodeficits. Microglial elimination promoted leukocyte infiltration and inflammatory cytokine levels in the ischemic brain. Of note, the exacerbated stroke severity induced by removal of microglia did not entirely depend on monocytes and lymphocytes infiltration. Importantly, microglial elimination dramatically augmented the production of inflammatory mediators by astrocytes after ischemia. We found that microglia restricted the ischemia-induced astrocyte response to confer neuroprotection. These findings suggest that the neuroprotective effect of microglia may result from its inhibitory action on the astrocyte response, implying that microglia may be central for neuron-astrocyte crosstalk in ischemic brain injury.

Microglial activation occurs very early after brain ischemia. Because microglia have multiple capabilities including phagocytosis, production of inflammatory or anti-inflammatory cytokines and antigen presentation, their presiding impact on brain injury and underlying mechanisms are not entirely clear.^55^ The debates regarding the possible detrimental or protective role of microglia in stroke are likely due to multiple factors including discrepancies in ways to deplete microglia and stroke models used in those studies.^15,16,18,19,56–58^ Despite a few studies showing that microglia may promote neural injury after stroke,^16,55,57^ growing evidence supports a protective role of microglia.^18,58^ In particular, one recent study demonstrated that elimination of microglia via PLX3397 enhanced ischemic brain injury by promoting neuronal excitoxicity.^39^ In line with present study, we found that microglial elimination favors increased brain infarct size and augments neurodeficits. However, it is important to note that none of these previous studies determined the impact of microglial elimination on brain inflammation and its contribution to ischemic brain injury.

As the most abundant cell type in the brain, astrocytes contribute to the regulation of neural transmission, survival of neurons and other glia cells, and the integrity of the blood–brain barrier under physiological conditions.^59,60^ In addition to these properties, astrocytes acquire the capacity for producing pro-inflammatory factors and antigen-presentation upon brain ischemia.^61,62^ This suggests the possibility that activation of astrocyte may promotes ischemic brain injury. Indeed, evidence shows that inhibition of astrocytes correlates with decreased infarct size and that treatments that decrease infarct size are often accompanied by attenuated astrocyte responses.^63,64^ In support of these findings, we found dramatically increased production of inflammatory factors by astrocytes after ischemia. Of interest, our in vitro studies show that microglia can suppress astrocyte production of inflammatory factors after ischemia. We therefore postulate that microglia may provide neuroprotection by suppressing the ischemia-induced astrocyte response. Nevertheless, still unclear are the mechanisms by which microglia may inhibit astrocyte activity in the setting of brain ischemia. This aspect awaits further investigations in future studies.

Our data cannot conclude that modulation of the astrocyte response is the only pathway that contributes to the protective role of microglia after brain ischemia. Although genetic deletion of lymphocytes or blockade of the homing of monocytes into the brain failed to prevent microglial elimination-induced exacerbation of stroke severity, we observed increased leukocyte infiltration after microglial elimination. Together with numerous studies showing the intimate crosstalk between brain-infiltrating leukocytes and microglia, whether and how microglia-leukocyte interaction may influence brain injury requires further studies. On the other hand, there is a possibility that microglia may exert protection via directly modulating neuronal activity after ischemia. As previously mentioned, a recent study shows that microglia elimination enhances excitotoxic neuronal injury,^39^ but how microglia could modulate the activity of ischemic neurons remains elusive. Our data show that microglia-conditioned media can reduce ischemia-induced neural damage as reflected by LDH release, suggesting the possibility that microglia-derived factors may be beneficial for ischemic neurons. Still unclear are whether microglia-derived factors can inhibit excitatory activity of ischemic neurons and what specific factors may be responsible. These aspects will certainly need to be investigated in future studies.

In addition to brain-resident microglia, other myeloid cells including monocytes and macrophages also express CSF1R. Therefore, there is a possibility that depletion of microglia may impact the baseline immune responses that contribute to the detrimental effect after brain ischemia. However, accumulating evidence shows that elimination of microglia had no discernable impact on neurological function and baseline brain inflammatory status under physiological conditions.^65,66^ In agreement with these findings, we found that PLX3397 treatment does not alter inflammation status, and only has minimal effects on monocytes and macrophages in the periphery in physiological condition (Figures 1 and 3). Together, these findings support that the detrimental effect of PLX3397 is caused by the removal of microglia. Of note, although we and others show that PLX3397 treatment has no significant impact on neurological function (Figure 1),^34^ we cannot conclude that the observed impact of PLX3397 in this study entirely results from CSF1R inhibition. The potential involvement of off-target effects cannot be excluded. Future studies using more selective CSF1R inhibitors may allow us to precisely dissect out the role of selective CSF1R inhibition in ischemic brain injury.

In conclusion, our data reveal a neuroprotective role of microglia after brain ischemia. The protection conferred by microglia may result from their inhibitory action on astrocyte response, implying that microglia may be central for neuron-astrocyte crosstalk in ischemic brain injury.

## Supplementary Material

Supplementary material
