# Supplementary material for: Depletion of microglia exacerbates postischemic inflammation and brain injury
Source: J Cereb Blood Flow Metab. 2017 Jan 1;37(6):2224–36. doi: 10.1177/0271678X17694185 (PMC5444553; doi:10.1177/0271678X17694185)
Supplement: Supplementary material [file JCB694185_supplementary_material.pdf]

Supplementary Materials

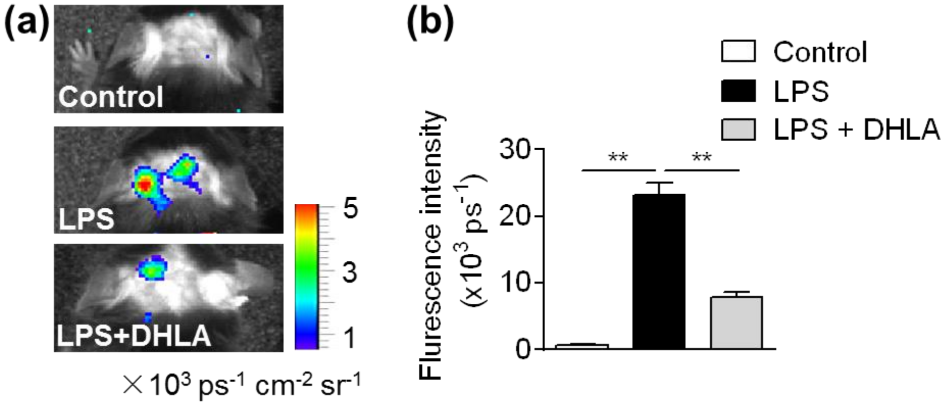

Supplementary Figure 1. Effect of DHLA on LPS-induced ROS production. Wild type mice received a single i.p. injection of 100 ng/mouse lipopolysaccharide (LPS), or 100 ng/mouse LPS plus 12.5 μm/mouse DHLA (dihydrolipoic acid, reduced form of α-lipoic acid). Wild type mice injected with phosphate buffered solution (PBS) were used as a control. ROS production was monitored by Xenogen IVIS200 imager using luminol (200 mg/kg, i.p.). **(a)** Representative bioluminescence images show ROS generation in mice receiving LPS, LPS plus DHLA or control PBS. **(b)** Summarized results show luminol signal intensity in mice receiving LPS, LPS plus DHLA or control PBS. n = 5 mice per group. Error bars represent SD; *P < 0.05; **P < 0.01.
